# Diagnosis of Prostate Cancer via Transanal Endoscopic Ultrasound–Guided Fine-Needle Biopsy in a Patient With Bone Lesions and a Rapid Clinical Response to Enzalutamide Plus Degarelix

**DOI:** 10.7759/cureus.100520

**Published:** 2025-12-31

**Authors:** Akinori Sasaki, Tomohiro Yamaba, Rika Kimura

**Affiliations:** 1 Gastroenterology, Tokyo Bay Urayasu Ichikawa Medical Center, Urayasu, JPN

**Keywords:** bone lesions, cancer of unknown primary, endoscopic ultrasonography-guided fine needle biopsy, enzalutamide plus degarelix, prostate cancer

## Abstract

In patients presenting with bone lesions suggestive of metastatic prostate cancer, rapid acquisition of diagnostic tissue is essential. Although transrectal ultrasound-guided (TRUS) or transperineal biopsy is standard, these approaches may be impractical in certain clinical settings. Markedly elevated serum prostate-specific antigen (PSA) levels can strongly suggest a prostatic origin, but histological confirmation remains necessary to initiate androgen receptor signaling inhibitor (ARSI)-based therapy. A 67-year-old man presented with diffuse arthralgia, impaired ambulation, and widespread osteosclerotic bone lesions on CT. Serum PSA was markedly elevated (5,230 ng/mL). Pelvic MRI could not be performed because of severe claustrophobia, and TRUS/transperineal biopsy was not feasible due to the absence of urology services at our institution. Bone biopsy was considered but judged less feasible than an endoscopic approach. Transanal endoscopic ultrasound (EUS) revealed a hypoechoic lesion in the right prostatic lobe, and EUS-guided fine-needle biopsy (EUS-FNB) using a 19-gauge needle yielded core tissue demonstrating PSA-positive adenocarcinoma. Despite an initial performance status (PS) of 4, driven primarily by pain, enzalutamide plus degarelix was initiated. The patient experienced rapid improvement in mobility and pain, with PSA falling to 11.3 ng/mL at two months and stable bone lesions on imaging. He returned to work in three months. This case illustrates that transanal EUS-FNB can provide a minimally invasive, feasible, and timely diagnostic option for patients in whom conventional prostate biopsy routes are impractical. Early histologic confirmation enabled prompt initiation of ARSI-based therapy, resulting in rapid symptomatic and biochemical improvement. EUS-FNB may be considered in selected cases of suspected prostate cancer when alternative biopsy approaches are not feasible.

## Introduction

Prostate cancer is among the most prevalent malignancies in men and frequently presents with osteosclerotic bone involvement in the metastatic setting [1]. Although the combination of diffuse bone lesions and markedly elevated prostate-specific antigen (PSA) levels suggests prostate cancer with high probability, histopathologic confirmation remains indispensable, particularly prior to initiating androgen receptor signaling inhibitor (ARSI)-intensified therapy [2, 3].

When standard routes such as transrectal ultrasound-guided (TRUS) or percutaneous biopsy are not feasible or prove non-diagnostic, alternative access strategies may be required [4]. Endoscopic ultrasound (EUS) enables a transrectal approach to pelvic lesions. Early feasibility work and subsequent multicenter experience suggest that EUS-guided fine-needle aspiration/biopsy (EUS-FNA/B) can safely sample extra-rectal pelvic masses and nodes with a high diagnostic yield, and isolated case reports have documented prostate cancer diagnosed via EUS-guided transrectal sampling [5-7]. Nevertheless, reports in which transanal EUS-FNB directly established a prostate primary during the initial evaluation of bone-predominant cancer of unknown primary and promptly guided therapy remain scarce.

For unresectable/metastatic prostate cancer, androgen-deprivation therapy (ADT) has long been the therapeutic backbone. Over the past decade, intensification from the hormone-sensitive phase with ARSIs has become standard of care: enzalutamide, abiraterone acetate, and apalutamide improved survival when added to ADT; more recently, darolutamide added to ADT plus docetaxel (ARASENS) further prolonged overall survival [8-11]. Because ARSI-based regimens generally have a more favorable tolerability profile than cytotoxic chemotherapy, their use may be considered even in patients with compromised performance status (PS), provided comorbidities and drug-specific toxicities are carefully assessed [12]. In fact, it has been reported that cancer-related pain can improve when patients with prostate cancer are treated with regimens that include ARSIs [13].

Here, we describe a patient with bone lesions, markedly elevated PSA, and inability to undergo conventional prostate biopsy, in whom transanal EUS-FNB enabled rapid histologic confirmation of prostate adenocarcinoma. This case underscores how tailoring the diagnostic access route can expedite both early diagnosis and early treatment, and we further discuss the potential utility of EUS-FNB in non-gastrointestinal malignancies.

## Case presentation

**Figure 1:**
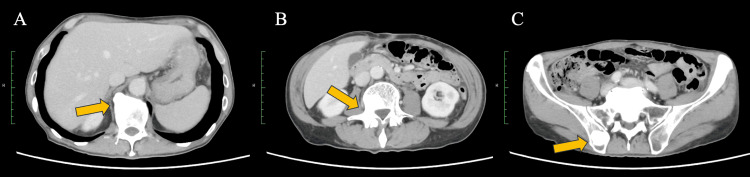
Baseline CT at presentation (A, B, C): The CT shows multiple sclerotic bone lesions involving the vertebral bodies and pelvic bones (arrows).

The patient was a 67-year-old man with a history of hypertension. Over the preceding month, he had developed diffuse arthralgia and loss of appetite, with progressive worsening to the point that ambulation became difficult, prompting admission to a referring hospital. Contrast-enhanced whole-body CT revealed disseminated metastatic bone lesions involving the vertebrae and pelvic bones, without an identifiable primary tumor (Figure 1). He was transferred to our institution for further work-up and treatment.

**Figure 2:**
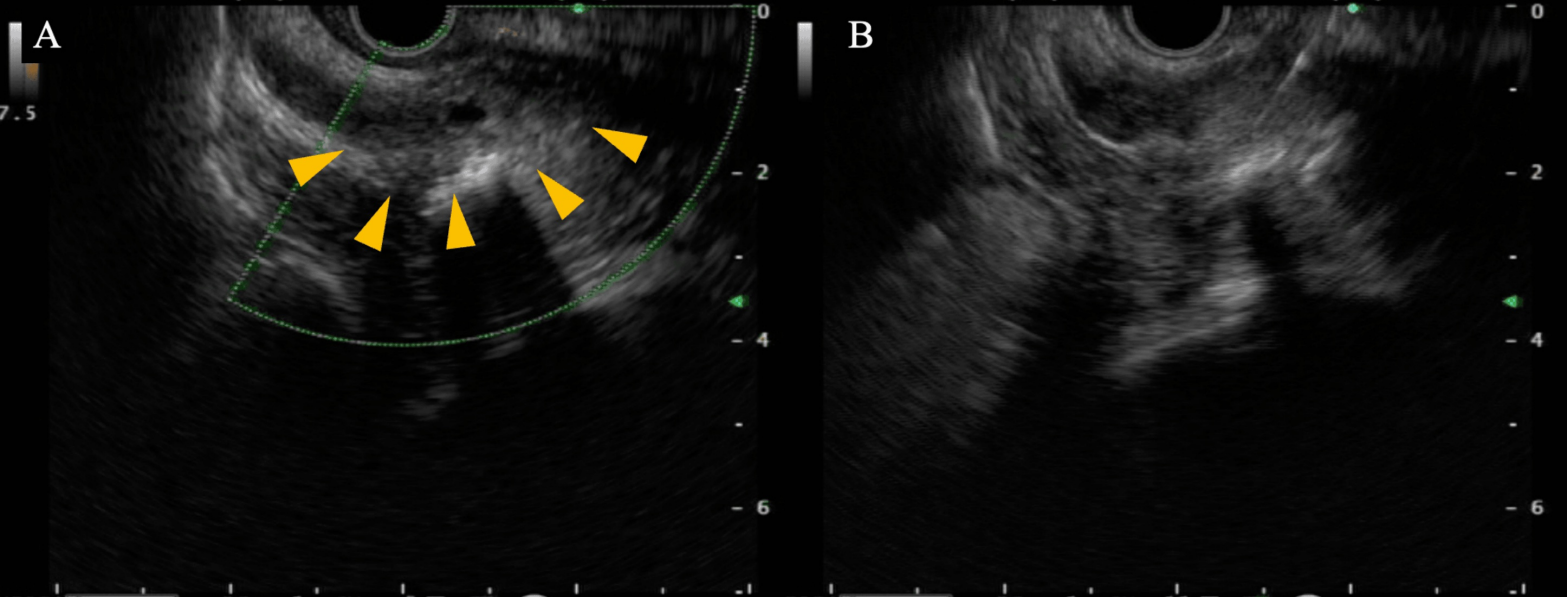
Transanal EUS and EUS-FNB Imaging (A) Transanal EUS identified a right-lobe prostatic lesion with focal necrosis (arrowheads). (B) The EUS-suspected prostatic lesion was punctured using an Acquire 19-gauge needle (Boston Scientific Corporation, Marlborough, MA, USA). EUS: endoscopic ultrasound; FNB: fine-needle biopsy

On admission, serum PSA was 5,230 ng/mL (reference range, 0-4 ng/mL), raising suspicion for a prostatic primary. However, pelvic MRI could not be performed because the patient had severe claustrophobia and was unable to tolerate the procedure. The absence of an MRI limited further non-invasive evaluation of the prostate. In our institution, no urologist was available, and standard prostate biopsy approaches such as TRUS-guided or transperineal biopsy were not feasible. Although bone biopsy was also considered, the bone lesions were located deep within the vertebrae and pelvic bones, and, given the patient’s severe pain and inability to ambulate, it was difficult to maintain a safe position for percutaneous biopsy. In contrast, transanal EUS-FNB was substantially less invasive and could be performed immediately, and this approach was therefore selected. On hospital day 2, EUS demonstrated an irregular, hypoechoic mass in the right prostatic lobe consistent with malignancy (Figure 2). We proceeded to EUS-FNB of the lesion using an Acquire 19-gauge needle (Boston Scientific Corporation, Marlborough, MA, USA). Two passes were made under conscious sedation with intravenous midazolam and pethidine. Adequate core samples were obtained without procedural complications. Histopathology revealed adenocarcinoma, and immunohistochemistry was positive for PSA, establishing a diagnosis of prostate cancer.

Although his PS was 4, this was attributed to severe bone pain rather than systemic organ failure; he was therefore considered a candidate for hormonal therapy. On hospital day 10, we initiated enzalutamide 160 mg once daily plus degarelix (loading 240 mg, then 80 mg every four weeks). Denosumab was started in parallel for bone metastasis-related pain and skeletal protection. The treatment course was uneventful, with no apparent adverse events attributable to enzalutamide or degarelix. Pain improved progressively (with concomitant analgesics), and by two weeks after treatment initiation, he was able to ambulate without pain and was discharged home.

**Figure 3:**
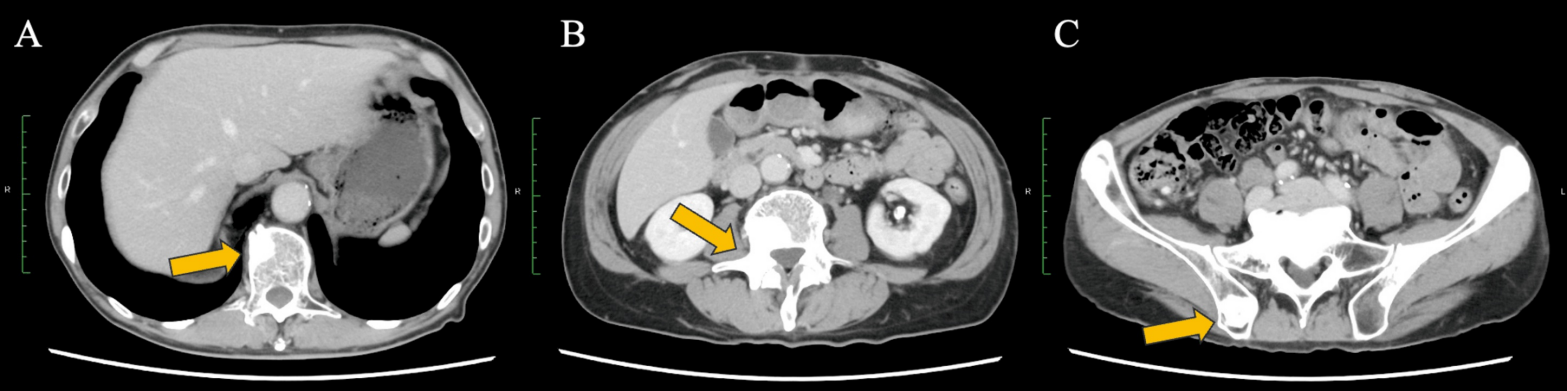
CT image obtained after treatment with enzalutamide plus degarelix (A, B, C): Post-treatment CT demonstrated persistent bone lesions without radiologic worsening (arrows) and no new sites of metastasis.

Outpatient therapy was continued. At two months, the PSA had decreased markedly (PSA, 11.3ng/ml), and contemporaneous chest-abdominal CT demonstrated no radiologic worsening of osseous disease and no new sites of metastasis (Figure 3). At three months, there was no evidence of disease progression, the patient’s quality of life had improved, and he had returned to work.

## Discussion

In this case, in a patient presenting with multifocal bone lesions and suspected metastatic prostate cancer, performing transanal EUS-FNB via the gastrointestinal route enabled a definitive diagnosis and facilitated prompt initiation of therapy. In patients with bone-predominant disease in whom prostate cancer is strongly suspected, imaging can be non-specific, and performance decline, bleeding or infection risk, or logistical issues may render TRUS-guided or percutaneous biopsy impractical. Leveraging EUS, originally refined for gastrointestinal indications, allowed a short-distance, high-resolution approach from the rectal side to the target, yielding high-quality core tissue with a 19-G FNB needle. Prior studies suggest that EUS-FNA/B can safely and effectively sample extrarectal pelvic masses and nodes, supporting its role as an alternative access route in selected prostate-area lesions when conventional routes are unsuitable [5, 7, 14].

Therapeutically, we initiated enzalutamide plus ADT (GnRH antagonist) despite a PS of 4, reasoning that PS deterioration was largely driven by pain and immobility from bone disease rather than irreversible organ dysfunction. In such scenarios, ARSI-based intensification of hormonal therapy can be a pragmatic option given its generally favorable tolerability compared with cytotoxic chemotherapy and its consistent survival and symptomatic benefits in randomized trials [8, 10, 15, 16]. Regarding pain, trials of enzalutamide have shown clinically meaningful benefits within approximately 12 to 13 weeks, including higher rates of pain palliation and delayed pain progression versus control [13]. Our patient’s early recovery of ambulation by week 2 and deep PSA decline by month 2 exemplify the coupling of tumor control with rapid symptomatic improvement that can occur in real-world practice [15, 16].

EUS-guided tissue acquisition has long been reported to be effective in gastrointestinal cancers, particularly pancreatic cancer and cholangiocarcinoma [17, 18]. By replacing percutaneous or open surgical approaches with EUS, these evaluations can be performed with reduced patient burden. Moreover, switching the device from EUS-FNA to EUS-FNB enables procurement of larger, architecturally preserved core specimens, which are often sufficient for immunohistochemistry and genomic testing [19].

Although EUS-FNB was initially used mainly for the diagnosis of gastrointestinal cancers, its simplicity and utility have led to increasing reports of diagnostic applications in non-gastrointestinal malignancies as well [7, 20]. As with gastrointestinal cancer indications, careful real-time confirmation under EUS guidance that no vessels or bowel lie along the needle trajectory can mitigate the risks of bleeding and gastrointestinal perforation when performing EUS-FNB for non-gastrointestinal cancers.

In the present case, EUS/EUS-FNB not only enabled direct evaluation of the prostate as the suspected primary site but also allowed sequential biopsy and on-site diagnostic confirmation within a single procedural pathway. Importantly, the use of EUS-FNB facilitated initiation of appropriate antitumor therapy by day 10 after referral to our institution.

Nevertheless, this case has limitations. In this patient with suspected metastatic prostate cancer, EUS was selected for primary-site evaluation and tissue acquisition in the context of limited access to standard urological biopsy techniques. In most centers, prostate tissue sampling is routinely performed via transperineal or transrectal routes under urological care, and the present experience with EUS-FNB may therefore not be generalizable. Moreover, because this is a single case report, it is not possible to determine the relative advantages or risks of EUS-FNB compared with conventional approaches; additional cases and comparative studies are required to clarify the specific clinical scenarios in which EUS-FNB offers the greatest benefit.

## Conclusions

Transanal EUS-FNB provided a minimally invasive, rapid, and feasible method of obtaining diagnostic tissue in a patient with suspected prostate cancer in whom standard biopsy routes were impractical. Histologic confirmation enabled prompt initiation of enzalutamide plus degarelix, resulting in rapid symptomatic and biochemical improvement. Although this is a single case, EUS-FNB may be considered as an alternative diagnostic approach when conventional biopsy methods are not feasible.
